# Toxicity of Amphotericin B Deoxycholate-Based Induction Therapy in Patients with HIV-Associated Cryptococcal Meningitis

**DOI:** 10.1128/AAC.01698-15

**Published:** 2015-11-17

**Authors:** Tihana Bicanic, Christian Bottomley, Angela Loyse, Annemarie E. Brouwer, Conrad Muzoora, Kabanda Taseera, Arthur Jackson, Jacob Phulusa, Mina C. Hosseinipour, Charles van der Horst, Direk Limmathurotsakul, Nicholas J. White, Douglas Wilson, Robin Wood, Graeme Meintjes, Thomas S. Harrison, Joseph N. Jarvis

**Affiliations:** a Institute of Infection and Immunity, St. George's University of London, London, United Kingdom; b Faculty of Epidemiology and Population Health, London School of Hygiene and Tropical Medicine, London, United Kingdom; c Elisabeth Hospital, Tilburg, the Netherlands; d Mbarara University of Science and Technology, Mbarara, Uganda; e University of North Carolina Project, Lilongwe, Malawi; f Mahidol-Oxford Research Unit, Faculty of Tropical Medicine, Mahidol University, Bangkok, Thailand; g Centre for Tropical Medicine and Global Health, Nuffield Department of Clinical Medicine, University of Oxford, Oxford, United Kingdom; h Edendale Hospital, Pietermaritzburg, South Africa; i Desmond Tutu HIV Centre, Institute of Infectious Disease and Molecular Medicine, University of Cape Town, Cape Town, South Africa; j Division of Infectious Diseases and HIV Medicine, Department of Medicine, University of Cape Town, Cape Town, South Africa; k Institute of Infectious Disease and Molecular Medicine, University of Cape Town, Cape Town, South Africa; l Department of Clinical Research, Faculty of Infectious and Tropical Diseases, London School of Hygiene and Tropical Medicine, London, United Kingdom; m Botswana-UPenn Partnership, Gaborone, Botswana; n Division of Infectious Diseases, Department of Medicine, Perelman School of Medicine, University of Pennsylvania, Philadelphia, Pennsylvania, USA

## Abstract

Amphotericin B deoxycholate (AmBd) is the recommended induction treatment for HIV-associated cryptococcal meningitis (CM). Its use is hampered by toxicities that include electrolyte abnormalities, nephrotoxicity, and anemia. Protocols to minimize toxicity are applied inconsistently. In a clinical trial cohort of AmBd-based CM induction treatment, a standardized protocol of preemptive hydration and electrolyte supplementation was applied. Changes in blood counts, electrolyte levels, and creatinine levels over 14 days were analyzed in relation to the AmBd dose, treatment duration (short course of 5 to 7 days or standard course of 14 days), addition of flucytosine (5FC), and outcome. In the 368 patients studied, the hemoglobin levels dropped by a mean of 1.5 g/dl (95% confidence interval [CI], 1.0 to 1.9 g/dl) following 7 days of AmBd and by a mean of 2.3 g/dl (95% CI, 1.1 to 3.6 g/dl) after 14 days. Serum creatinine levels increased by 37 μmol/liter (95% CI, 30 to 45 μmol/liter) by day 7 and by 49 μmol/liter (95% CI, 35 to 64μmol/liter) by day 14 of AmBd treatment. Overall, 33% of patients developed grade III/IV anemia, 5.6% developed grade III hypokalemia, 9.5% had creatinine levels that exceeded 220 μmol, and 6% discontinued AmBd prematurely. The addition of 5FC was associated with a slight increase in anemia but not neutropenia. Laboratory abnormalities stabilized or reversed during the second week in patients on short-course induction. Grade III/IV anemia (adjusted odds ratio [aOR], 2.2; 95% CI, 1.1 to 4.3; *P* = 0.028) and nephrotoxicity (aOR, 4.5; 95% CI, 1.8 to 11; *P* = 0.001) were risk factors for 10-week mortality. In summary, routine intravenous saline hydration and preemptive electrolyte replacement during AmBd-based induction regimens for HIV-associated CM minimized the incidence of hypokalemia and nephrotoxicity. Anemia remained a concerning adverse effect. The addition of flucytosine was not associated with increased neutropenia. Shorter AmBd courses were less toxic, with rapid reversibility.

## INTRODUCTION

Amphotericin B (AmB) deoxycholate (AmBd) is a polyene antifungal that binds to ergosterol in the fungal cell membrane, resulting in increased permeability and cell death (1) and binds to cholesterol in mammalian cell membranes, partly explaining its adverse effects of nephrotoxicity, electrolyte imbalance, and anemia (1).

Less toxic lipid formulations of AmB, developed in the 1990s, have now largely supplanted the use of the AmBd preparation for the treatment of systemic mycoses in high-resource settings. AmBd has been used to treat cryptococcal meningitis (CM) since the pre-HIV era (2, 3). AmB exhibits concentration-dependent killing (4), and successive randomized controlled trials used progressively increasing doses as induction therapy for HIV-associated CM, with or without flucytosine (5FC) or fluconazole as a second agent (5−9). Treatment with AmBd at a dose of 1 mg/kg of body weight/day plus 5FC provided the best fungal clearance and 10-week survival rates in a recent phase III trial (10) and remains the “gold standard” for the treatment of HIV-associated CM according to IDSA and WHO guidelines (11, 12).

AmBd nephrotoxicity results from decreased renal blood flow resulting in a reduced glomerular filtration rate and direct renal tubular toxicity, causing potassium and magnesium losses (1). Nephrotoxicity is cumulative and dose dependent although reversible (13, 14) and is reduced by preloading with saline, adequate hydration, and electrolyte replacement (15−19). Anemia results primarily from inhibition of renal erythropoietin production, although hemolysis has been reported (20).

In resource-poor settings, facilities to prevent, monitor, and manage AmBd toxicity are lacking. Fear of toxicities deters clinicians from using AmBd to treat CM. Instead, less effective but widely available fluconazole monotherapy is used (21).

Since 2000, we have implemented a standardized protocol of prehydration and preemptive electrolyte supplementation in clinical trials of AmBd-based CM induction treatment in Asia and Africa (14, 22–27), which is now included in WHO guidelines (12). We present toxicity analyses of AmBd administered at a dose in the currently recommended dose range (0.7 to 1 mg/kg/day) for treatment durations of 5 to 14 days, alone or combined with a second antifungal, in a large combined cohort of patients with HIV-associated CM managed in resource-limited settings. Our aims were to characterize the association of AmBd dose and duration, and the addition of 5FC, with the development of its common toxicities and to determine the relationship between toxicity and mortality.

## MATERIALS AND METHODS

### Participants and procedures.

The cohort consisted of adult HIV-infected, antiretroviral therapy (ART)-naive patients with a first episode of CM, enrolled in six phase II clinical trials carried out from 2002 to 2010 in Asia and Africa (14, 23–27) (see Table S1 in the supplemental material). All trials were approved by the Research Ethics Committees at each site and at St. George’s University of London.

In trials 1 to 4, patients received standard (14-day) courses of AmBd, and in studies 5 and 6, patients received shorter courses (≤7 days). Induction therapy differed in terms of the AmB dose (0.7 or 1 mg/kg/day, dosed according to actual body weight), choice of second and/or third drug, and adjunctive gamma interferon. All patients on 5FC received 5FC orally, except for 32 patients in the Thai study, who received intravenous (i.v.) 5FC as part of a pharmacokinetic substudy (28).

### Management of toxicity. (i) Laboratory monitoring.

During the first 14 days, measurements of complete blood counts and urea, creatinine, and electrolyte levels were performed for all patients on alternate days (measurement of magnesium levels was available only in South Africa).

### (ii) Electrolytes and renal impairment.

Patients received daily prehydration with 1 liter 0.9% (normal) saline with 20 mmol potassium chloride (KCl) before AmBd administration. Additional i.v. fluids were administered at the study physicians' discretion. Nephrotoxic drugs such as aminoglycosides and nonsteroidal anti-inflammatory drugs (NSAIDs) were avoided (premedication with paracetamol or chlorpheniramine was allowed in the case of infusion reactions). If the creatinine level rose to 220 μmol/liter (2.5 mg/dl), equivalent to grade III nephrotoxicity based on Division of AIDS (DAIDS) criteria (creatinine increase of 1.9 to 3.4 times the upper limit of normal [ULN]), the next AmBd dose was omitted, and the patient was given additional i.v. fluids. The following day, if the creatinine level was stable or improving, alternate daily dosing was instituted at the same AmBd dose, with a return to daily dosing once the creatinine level had normalized. If the creatinine level was still increasing, AmBd treatment was stopped, and the patient was switched early to fluconazole, adjusting the dose according to renal function (50% for a creatinine clearance rate of 20 to 40 ml/min and 25% for a creatinine clearance rate of <20 ml/min). The flucytosine dose interval was extended to a 12-h interval if the creatinine clearance rate decreased to 20 to 40 ml/min and once daily if the creatinine clearance rate was <20 ml/min. In all South African trials, patients received oral electrolyte supplementation with 2 tablets of potassium chloride (Slow-K, 600 mg [8 mmol K/tablet]) twice daily and 2 tablets of magnesium chloride (Slow-Mag, 535 mg [5.33 mmol Mg/tablet]) once daily, with additional i.v. or oral supplementation as required. At other sites, additional i.v. or oral potassium was administered according to laboratory results.

### (iii) Bone marrow toxicity.

Due to limited blood product availability, there was no hemoglobin (Hb) threshold for transfusion; this was done at the study sites' discretion. Flucytosine treatment was stopped if platelet numbers dropped to <50 × 10^9^ platelets/ml or if neutrophil numbers dropped to <0.5 × 10^9^ neutrophils/liter.

### Statistical analyses.

We explored changes in Hb levels, neutrophil and platelet numbers, creatinine levels, K levels, and Mg levels over a 14-day period following the start of AmBd-based treatment. For each measure, we used LOESS (locally weighted scatterplot smoothing) to estimate the mean value as a function of the number of days after treatment initiation.

Linear regression models were constructed to examine the effects of the AmB dose (0.7 or 1 mg/kg/day) and treatment duration (5 to 7 days or 14 days) on changes in Hb, K, and creatinine levels from baseline to days 7 and 14; peak creatinine levels; and Hb nadir. Sex and treatment with 5FC were included as potential confounders in Hb analyses. Baseline imbalances between groups were accounted for by using a “change from baseline” analysis rather than adjusting for baseline (with the exception of peak and nadir values).

Logistic regression models examined associations between protocol-predefined nephrotoxicity, DAIDS grade III hypokalemia (K^+^ level of 2 to 2.4 mmol/liter or meq/liter), grade III (Hb level of 6.5 to 7.4 g/dl) and grade IV (Hb level of <6.5 g/dl) anemia, and mortality at 2 and 10 weeks, with adjustments for baseline values (Hb, creatinine, and K), patient weight in the nephrotoxicity analysis, and the established prognostic markers baseline fungal burden, altered mental status, and CD4 cell count (29).

Given the pooling of data across studies, clustering by study in both the linear and logistic regression models was accounted for by including “study” as a random-effect term.

Data were analyzed by using Stata, v12.0 (StataCorp, TX, USA).

## RESULTS

**FIG 1:**
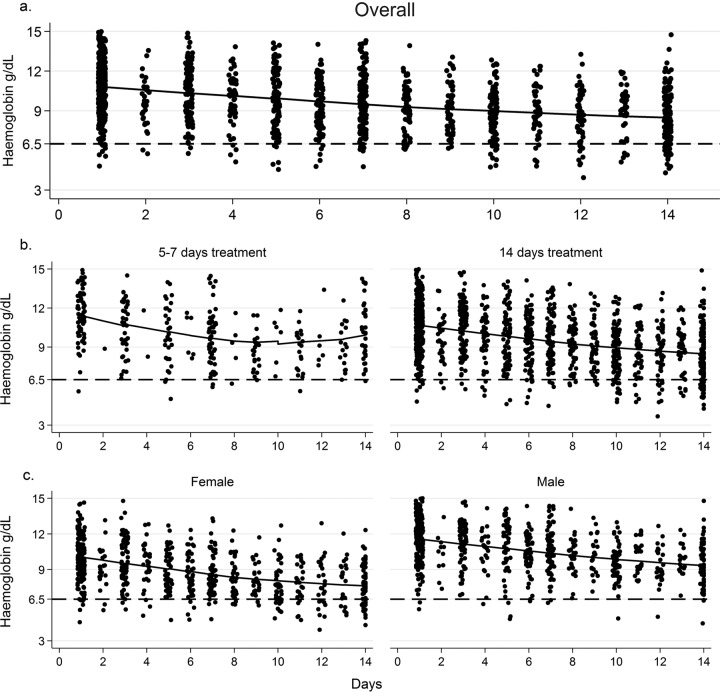
Individual data points and fitted LOESS curves for hemoglobin values over the first 14 days of antifungal therapy. (a) All patients receiving 14 days of AmB-based induction therapy. (b) Plot by AmB duration (short-course versus standard treatment). (c) Plot by sex. The broken line indicates a DAIDS grade IV adverse-event threshold of 6.5 g/dl.

**FIG 2:**
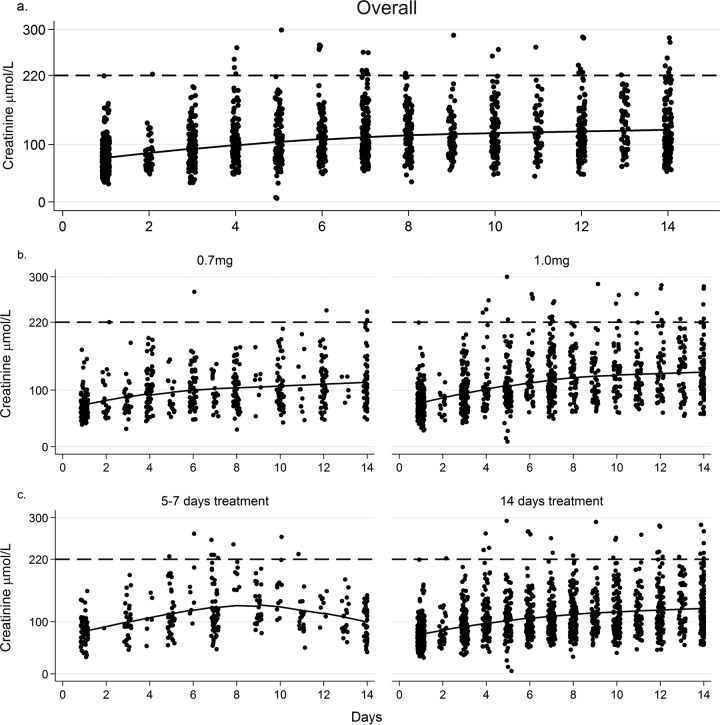
Individual data points and fitted LOESS curves for creatinine values over the first 14 days of antifungal therapy. (a) All patients receiving 14 days of AmB-based induction therapy. (b) Plot by AmB dose (0.7 versus 1 mg/kg/day). (c) Plot by AmB duration (short-course versus standard treatment). The broken line indicates a threshold of 220 μmol/liter (protocol definition of nephrotoxicity).

A total of 368 (52% male) patients were included: 64 (17%) were Thai, 298 (81%) were black African, and 3 (<1%) were of mixed race. The median age was 33 years (interquartile range [IQR], 29 to 38 years), and the CD4 count was 25 cells/μl (IQR, 10 to 55 cells/μl). Changes from baseline (pretreatment) are shown in Table 1, and LOESS curves are shown in Fig. 1 and 2.

**TABLE 1:** Changes in laboratory parameters from baseline values over 1 and 2 weeks of AmBd treatment^*a*^

Laboratory parameter	Mean value (95% CI)				
	Baseline value	Absolute change to day 7	% change to day 7	Absolute change to day 14^*b*^	% change to day 14^*b*^
Hemoglobin level (g/dl)	11 (10.4, 11.6)	−1.5 (−1.9, −1)	−12 (−16, −9)	−2.3 (−3.6, −1.1)	−20 (−32, −8)
Creatinine level (μmol/liter)	77 (68,87)	+37 (30, 45)	+52 (43, 62)	+49 (35, 64)	+73 (53, 93)
Potassium level (mmol/liter)	3.9 (3.8, 4.0)	+0.2 (−0.0, 0.5)	+9 (3, 15)	+0.1 (−0.1, 0.3)	+6 (−1, 11)
Magnesium level (mmol/liter)	0.7 (0.6, 0.8)	−0.07 (−0.12, −0.02)	−1(−7, 5)	−0.15 (−0.2, −0.1)	−10 (−33, −13)
Neutrophil count (10^9^/liter)	3.5 (2.2, 4.9)	−0.1 (−0.4, 0.0.1)	+8 (0.9, 14)	−0.3 (−0.5, 0.0)	+8 (−3, 20)

a Values shown are means with 95% CIs adjusted for study-level clustering.

b Changes from baseline to day 14 are calculated only for patients enrolled in studies of standard-course AmBd treatment(1–4).

From a mean baseline of 11 g/dl, the mean overall drops in hemoglobin levels were 1.5 g/dl (12%) by day 7 and 2.3 g/dl (20%) by day 14. With preemptive supplementation, potassium values remained stable over time (see Fig. S1a in the supplemental material). The mean change in creatinine levels from baseline values were +37 μmol/liter (95% CI, 30 to 45 μmol/liter) at day 7 and +49 μmol/liter (95% CI, 35 to 64 μmol/liter) at day 14.

### Incidence of serious toxicity.

Grade III/IV anemia (Hb level of <7.5 g/dl) developed in 112 patients (33%).

Fifty-nine patients (18%) developed grade IV anemia (Hb level of <6.5 g/dl). Women had higher incidences of grade III (54% versus 14%) and grade IV (31% versus 5%) anemia (*P* < 0.001) due to sex differences in baseline values rather than differences in on-treatment change (day 14 drop in Hb level of 2.14 g/dl for males versus 2.21 g/dl for females; *P* = 0.92) (Fig. 1c). In the South African trials, which collected transfusion data, 12 of 304 patients (4%) received blood transfusions, constituting only 20% of patients with grade IV anemia.

Sixteen of 284 patients (5.6%) developed grade III hypokalemia (K^+^ level of <2.5 mmol/liter), 5 in the first week of treatment. Three patients (1.1% of the cohort) developed grade IV hypokalemia (K^+^ level of <2 mmol/liter): 2 on day 11 and 1 on day 12 of treatment. In the South African trials using preemptive oral in addition to i.v. K^+^ supplementations, the change in the potassium level from baseline differed significantly from those in trials not using oral K (mean day 7 change in the K level of +0.39 mmol/liter [95% CI, 0.24 to 0.54 mmol/liter] versus −0.17 mmol/liter [95% CI, −0.47 to +0.12 mmol/liter]; *P* < 0.001 by *t* test), with a lower incidence of grade III hypokalemia (4.2% versus 9.7%; *P* = 0.08).

Thirty-three patients (9.5%) exceeded the protocol-defined nephrotoxicity threshold (creatinine level of >220 μmol/liter). In the South African trials using standard-course AmBd (*n* = 234), 15 patients (6.4%) discontinued AmBd early due to nephrotoxicity, at a median of 11 days (range, 5 to 13 days). In the short-course trials (*n* = 70), 4 patients (5.7%) stopped treatment early (all in the 7-day study), 3 of whom omitted the final day only.

### Toxicity by AmB dose and duration.

There was a greater absolute drop in the Hb level at days 7 and 14 in patients receiving the higher AmBd dose and in those receiving standard treatment versus short-course treatment, although differences were nonsignificant (see Table S2 in the supplemental material). Patients receiving shorter courses had higher nadir Hb levels (mean of 9.5 versus 8.3 g/dl), even after adjustment for sex and 5FC treatment (*P* = 0.033).

There was less renal impairment in those receiving the 0.7-mg/kg AmBd dose, with mean increases in creatinine levels of 28 versus 41 μmol/liter at day 7 (*P* = 0.02) and 42 versus 53 μmol/liter at day 14 (*P* = 0.09). The mean creatinine level peaked at 127 versus 145 μmol/liter (*P* = 0.04), remaining significant upon multivariable analysis (adjusted mean difference of 13 μmol/liter; *P* = 0.04). Despite similarities in peak creatinine levels, the change in creatinine levels to day 14 was less in patients receiving short-course than in those receiving standard treatment (mean increase of 17 versus 49 μmol/liter; *P* < 0.001) (see Table S2 in the supplemental material).

### Reversibility.

The Hb level stabilized and started to increase in the second week in the short-course group after AmBd treatment was stopped, while it continued to decline in the standard-course group (mean change at week 2 of −0.12 versus −0.93 g/dl; *P* = 0.01) (Fig. 1b).

Renal impairment in the short-course group resolved in week 2 following cessation of AmBd, with the creatinine levels continuing to rise in the standard-course group (mean change at week 2 of −28 versus +14 μmol/liter; *P* < 0.001) (Fig. 2c).

### Potassium.

There was no significant association of AmB dose or duration with changes in potassium levels (data not shown). The LOESS curves of changes in potassium levels (see Fig. S1 in the supplemental material) show a dip in K^+^ levels during days 8 to 10 in the short-course trials, after AmBd administration and K^+^ supplementation were stopped, which is supportive of ongoing potassium wasting (30).

Incidences of grade III/IV hypomagnesemia, neutropenia, and thrombocytopenia and associations with the use of 5FC are described in the supplemental material.

### Association of toxicity with 2- and 10-week outcomes.

Development of nephrotoxicity (creatinine level of >220 μmol/liter) was associated with 2-week mortality (Table 2); however, this was no longer significant in the adjusted model (2-week adjusted odds ratio [aOR], 2.9; 95% CI, 0.6 to 12.7; *P* = 0.17). The association between nephrotoxicity and 10-week mortality remained significant following adjustment for the baseline creatinine level, weight, CD4 count, fungal burden, altered mental status, and study (10-week aOR, 4.5; 95% CI, 1.8 to 11; *P* = 0.001) (Table 2).

**TABLE 2:** Association between development of toxicities and 2- and 10-week mortality^*a*^

Toxicity variable and category	Outcome at 2 wk					Outcome at 10 wk				
	% mortality	OR (95% CI), univariable	*P* value for OR	aOR (95% CI), multivariable	*P* value for aOR	% mortality	OR (95% CI), univariable	*P* value for OR	aOR (95% CI), multivariable	*P* value for aOR
Nephrotoxicity^*b*^										
Creatinine peak level of >220 μmol/liter	21	3.1 (1.2–7.8)	0.018	2.8 (0.6–12.7)	0.17	53	4.2 (2–8.8)	<0.001	4.5 (1.8–11)	0.001
Creatinine peak level of <220 μmol/liter	8	1		1		21	1		1	
Anemia grade III/IV										
Hb nadir of <7.5 g/dl	6	0.8 (0.33–2)	0.65			31	2.1 (1.2–3.5)	0.008	2.2 (1.1–4.3)	0.028
Hb nadir of >7.5 g/dl	8	1				18	1		1	
Hypokalemia grade III										
K nadir of <2.5 mmol/liter	6	0.77 (0.1–6.1)	0.81			25	0.99 (0.3–3.2)	0.99		
K nadir of >2.5 mmol/liter	8	1				25	1			

a Adjusted for the baseline variables (Hb, creatinine, and K), baseline fungal burden, mental status, CD4 count, and study.

b Because of the association of weight with nephrotoxicity as well as outcome, weight was additionally adjusted for in the nephrotoxicity analysis.

Severe anemia (grade III/IV) was a significant risk factor for 10-week mortality following adjustment for the baseline Hb level, CD4 count, fungal burden, altered mental status, and study (aOR, 2.2; 95% CI, 1.1 to 4.3; *P* = 0.028) (Table 2).

Development of grade III hypokalemia (K^+^ level of <2.5 mmol/liter) was not associated with mortality (Table 2).

## DISCUSSION

This analysis of 368 patients treated with AmBd-based induction regimens in resource-poor settings represents the largest reported toxicity analysis of treatment for HIV-associated CM.

Trials reporting the toxicity of AmBd at the recommended 0.7- to 1-mg/kg/day doses for HIV-associated CM include 6 trials in high-income settings (6, 8, 31–34) and 8 trials in low-income settings in Asia and Africa (9, 10, 35–40) (see Table S3 in the supplemental material). Six trials reported routine use of saline preloading, and one reported preemptive electrolyte replacement (41). Definitions of nephrotoxicity varied. The incidence of grade III nephrotoxicity (or equivalent) was 20 to 33% in older trials without routine fluid preloading (8, 32, 36), compared to 2 to 11% in our study and in more recent trials using saline preloading (10, 39). Six percent of our patients discontinued treatment early, usually 1 to 2 days before completion, in comparison to up to 53% in previously reported trials.

AmBd nephrotoxicity is cumulative (42): in our patients, the creatinine level rose by a mean of 52% by day 7 and by a mean of 73% by day 14. Although nephrotoxicity was greater for the 1-mg/kg/day dose, this translated into a mean difference in the peak creatinine level of 13 μmol/liter, which is of minor clinical significance and should not deter clinicians from using the more-fungicidal, higher dose.

The long terminal half-life of AmBd (up to 2 weeks) (4) provides ongoing fungicidal activity after drug administration is stopped (25, 27). These data demonstrate better tolerability of 5- to 7-day regimens, with less nephrotoxicity and reversibility and with creatinine levels returning almost to baseline 1 week after discontinuation of short-course AmBd treatment. Ongoing electrolyte losses following AmBd discontinuation mean that supplementation and monitoring are needed for several more days, an important implementation issue, as stable patients are usually discharged following discontinuation of intravenous therapy. Short-course regimens minimize toxicity while potentially maintaining efficacy as well as reducing costs of hospitalization, intravenous administration, and laboratory monitoring. A phase III trial comparing short-course to standard AmBd regimens is under way in Africa (ACTA, ISRCTN Registry number 45035509). Administration of fewer, higher doses of liposomal preparations may be an alternative (AmBition-CM, ISRCTN Registry number 10248064).

The reported incidences of hypokalemia (7 studies, with variable definitions) (see Table S3 in the supplemental material) range from <1 to 56%. In this cohort, with routine preemptive supplementation with 20 mmol KCl (and supplemental oral potassium in South Africa), potassium levels changed little over 14 days. In a recent large Vietnamese trial (10), 18% of patients developed grade III hypokalemia, as opposed to only 6% in our cohort and 9% in a Ugandan cohort using preemptive replacement (41). These data support WHO recommendations for preemptive daily supplementation with 20 mmol KCl i.v., with additional oral supplementation (12).

Despite oral supplementation, hypomagnesemia was much more common than hypokalemia in our cohort. Unfortunately, magnesium levels were not measured at study sites where routine supplementation was not given, for comparison. The lack of availability of magnesium monitoring or oral replacement preparations (magnesium chloride or glycerophosphate) is a problem. i.v. magnesium (usually stocked for preeclampsia) can be considered for patients with severe deficiency or those remaining hypokalemic despite potassium replacement (12).

Rates of anemia reported in 8 studies (variable definitions) (see Table S3 in the supplemental material) ranged from 37 to 44%. Two trials reported an average drop in the Hb level of 1.5 to 2.5 g/dl; in one trial, 59% of patients received transfusions (34, 38). Despite the development of grade IV anemia (Hb level of <6.5 g/dl) in 16% of our cohort, with a mean decrease in hemoglobin levels of 2.3 g/dl over 14 days, only 4% of patients received transfusions, reflecting the scarcity of blood for transfusion in Africa. Severe AmBd-induced anemia is potentially life-threatening in such settings, given the low baseline Hb levels and the current lack of validated preventative interventions.

The addition of oral 5FC to AmBd treatment did not result in clinically significant excess toxicity in our cohort, with a slightly greater drop in the hemoglobin level but no increases in rates of neutropenia or thrombocytopenia, an important observation given that this combination remains the recommended CM induction regimen.

Development of nephrotoxicity and severe anemia were independent risk factors for mortality, with odds of dying at 10 weeks being increased 2-fold for patients with a decrease of the hemoglobin level of <7.5 g/dl and >4-fold in those with an increase of the creatinine level of >220 μmol/liter. Using these cutoffs, anemia was >3 times more common than nephrotoxicity. The lack of an association between hypokalemia and mortality is likely related to routine preemptive potassium replacement.

A limitation of this study is the pooling of data from multiple cohorts treated with different AmBd-based combination regimens from four countries on two continents over an 8-year period. Although toxicity management was uniform across trial sites, there may have been heterogeneities in clinical management. To address this, we adjusted for potential variable-specific and outcome-related confounders in the multivariate analyses and included an additional study variable to try to account for any residual confounding due to unmeasured effects related to temporal or site-specific differences.

Findings from this large cohort of patients treated in resource-poor settings demonstrate that with appropriate laboratory monitoring, it is possible to implement the most fungicidal AmBd-based induction treatment regimens for HIV-associated CM by using a standardized protocol for preemptive toxicity management. Hypokalemia was minimized, and nephrotoxicity occurred in <10% of patients. Anemia remains a concerning side effect. Shorter courses of AmBd are less toxic, with rapid reversibility. The implementation of standardized protocols for preemptive toxicity management is a priority in countries with a high HIV prevalence to maximize the benefits of a drug that is likely to remain a cornerstone of CM treatment for many years to come.

## Supplementary Material

Supplemental material
