# Supplementary material for: Toxicity of Amphotericin B Deoxycholate-Based Induction Therapy in Patients with HIV-Associated Cryptococcal Meningitis
Source: Antimicrob Agents Chemother. 2015 Nov 17;59(12):7224–31. doi: 10.1128/AAC.01698-15 (PMC4649151; doi:10.1128/AAC.01698-15)
Supplement: Supplemental material [file supp_59_12_7224__index.html]

Toxicity of Amphotericin B Deoxycholate-Based Induction Therapy in Patients with HIV-Associated Cryptococcal Meningitis — Supplemental material 

# Toxicity of Amphotericin B Deoxycholate-Based Induction Therapy in Patients with HIV-Associated Cryptococcal Meningitis

## Supplemental material

- Supplemental file 1 -

  Additional results, Supplemental Tables S1 to S3, and Figure S1.

  PDF, 707K
