# Supplementary material for: Toxicity of Amphotericin B Deoxycholate-Based Induction Therapy in Patients with HIV-Associated Cryptococcal Meningitis
Source: Antimicrob Agents Chemother. 2015 Nov 17;59(12):7224–31. doi: 10.1128/AAC.01698-15 (PMC4649151; doi:10.1128/AAC.01698-15)

Supplementary Results

Magnesium

Serial magnesium levels were monitored in 117 patients (Table 1). In contrast to potassium, the supplementation protocol was insufficient to prevent development of hypomagnesemia in a moderate number of patients: 29 patients (24.7%) developed grade III (0.3-0.44 mmol/L) and 7(5.9%) grade IV hypomagnesemia (<0.3 mmol/L).

Neutrophils

Serial neutrophil counts were performed in 213 patients. There was marked variability in change from baseline as some patients developed sepsis, accompanied by neutrophilia (Table 1). There were no significant differences in neutrophil change at day 7 or day 14 in patients treated with, or without, 5FC(mean change -0.11 versus -0.17 $x10^9/L$ at day 7, p=0.82; -0.36 v -0.46 $x10^9/L$ at day 14, p=0.79). No Thai patients developed grade IV neutropenia (neutrophils<$0.5x10^9/L$). Amongst the 149 African patients who had serial differential counts, 9 patients(6%) developed grade IV neutropenia during treatment, of whom only 5 were on 5FC. Five of 9 had reversal on subsequent laboratory testing. For the remaining 4 patients it was the last value available.

Inclusion of 5FC as the second drug led to slightly greater drop in Hb at day 7 and day 14 (0.46g/dL and 0.48g/dL greater, respectively, in analysis adjusted for sex and AmBd dose). This difference was significant at day 7 (95% CI 0.06, 0.85g/dL, p=0.023), but not at day 14 (95% CI -0.28, 1.23, p=0.22).

Platelets

11 of 325 patients (3.4%) developed Grade III/IV thrombocytopenia (platelet count $<50x10^9/L$). There were no significant differences in platelet change to day 14 for patients treated with, or without 5FC (mean change -33 vs $-1x10^9/L$, p=0.2).

Supplementary table 1. Numbers of patients receiving each treatment regimen (by AmBd dose, duration and second/third drugs) in each trial (1-6) included in the analysis

	1	2	3	4	5	6	Total *n*
**Amb 5d 1mg + Fluc 1200**	0	0	0	0	30	0	**30**
**Amb 7d 1mg + Fluc 1200**	0	0	0	0	0	20	**20**
**Amb 7d 1mg + Fluc 1200 + 5FC**	0	0	0	0	0	20	**20**
**Amb 14d 0.7mg**	16	0	0	0	0	0	**16**
**Amb 14d 0.7mg + 5FC**	16	30	5	0	0	0	**51**
**Amb 14d 0.7mg + Fluc 400**	16	0	0	0	0	0	**16**
**Amb 14d 0.7mg +Fluc 400 + 5FC**	16	0	0	0	0	0	**16**
**Amb 14d 1mg + 5FC**	0	34	16	31	0	0	**81**
**Amb 14d 1mg + Fluc 800**	0	0	22	0	0	0	**22**
**Amb 14d 1mg + Fluc 1200**	0	0	24	0	0	0	**24**
**Amb 14d 1mg + Vori**	0	0	13	0	0	0	**13**
**Amb 14d 1mg + 5FC + IFN2**	0	0	0	29	0	0	**29**
**Amb 14d 1mg + 5FC + IFN6**	0	0	0	30	0	0	**30**
**Total *n***	**64**	**64**	**80**	**90**	**30**	**40**	**368**

Interferon-γ was given as either 2 doses (days 1,3) or 6 doses (days 1,3,5,8,10,12) [25].
Individual study sites and protocols were as follows:
1) Thailand, randomized controlled trial (RCT) of 14 days' AmBd 0.7mg/kg either alone, or plus flucytosine(5FC) 100mg/kg/d, fluconazole 400mg/d, or fluconazole 400mg/d plus 5FC[24];
2) South Africa. RCT 14d AmB 0.7mg/kg/d versus AmB 1mg/kg/d, both with 5FC 100mg/kg/d[14];
3) South Africa. RCT 14d AmB 1mg/kg/d for 14 days plus either 5FC 100mg/kg/d, fluconazole 800mg/d, fluconazole 1200mg/d or voriconazole 300mg/bd[27];
4) South Africa. RCT 14d AmB 1mg/kg/d plus 5FC 100mg/kg/d, +/- adjunctive interferon-γ[25];
5) Uganda, cohort study of 5 days' AmB 1mg/kg/d plus fluconazole 1200mg/d[26]**;**
6) Malawi, RCT 7d AmB 1mg/kg/d plus either fluconazole 1200 mg/day for 14 days or fluconazole 1200 mg/day plus 5FC 100 mg/kg/d for 14 days (oral arm not included in this analysis) [28].

Supplementary Table 2. Univariable and Multivariable analyses of change in haemoglobin and creatinine by group

Variable	Category	Mean (95%CI)	Difference (beta coefficient, 95%CI)	p-value	Adjusted beta coefficient (95%CI)	Adjusted p-value[a]
**AmB dose (0.7 vs 1mg/kg/d)**						
**Hb drop 7**	0.7	1.2 (0.0, 2.3)				
	1	1.6 (1.2, 2.0)	0.4 (-0.1,0.9)	0.107	0.4(-0.1,0.8)	0.088
**Hb drop 14**	0.7	2.1 (0.1, 4.2)				
	1	2.4 (0.3, 4.4)	0.7 (-0.3,1.7)	0.174	0.7(-0.4,1.8)	0.208
**Creat rise 7**	0.7	28 (19, 37)				
	1	41 (34, 48)	13 (2, 23)	0.016		
**Creat rise 14**	0.7	42 (34,49)				
	1	53 (33, 74)	12 (-2, 25)	0.086		
**Creat peak**	0.7	127 (107,147)				
	1	145 (131, 159)	17 (1, 33)	0.038	13 (0.5,26)	0.042
**AmB duration (5-7 days vs 14 days)**						
**Hb drop 14**	5-7d	1.7(-7.0,10.4)				
	14d	2.3(1.1,3.6)	0.7 (-0.5,1.8)	0.243	0.5 (-0.5, 1.5)	0.313
**Hb nadir**	5-7d	9.5(-4.2,23.3)				
	14d	8.3(7.7,8.8)	-1.5(-2.6,-0.3)	0.012	-1.5(-2.8,-0.1)	0.033
**Creat rise 14**	5-7d	17 (2,33)				
	14d	49 (35,64)	32 (20, 45)	<0.001		
**Creat peak**	5-7d	135 (-114,385)				
	14d	140 (121,160)	6(-18, 30)	0.61	11 (-6 ,28)	0.216

Values shown are means with 95% confidence intervals adjusted for study-level clustering
Hb/Creat drop/rise 7 or 14= absolute change in Hb/Creat from day 1 to day 7 or 14
Hb nadir=lowest Hb over 14 days
Creat peak= highest Creat value over 14 days
Group comparisons by dose at 14 days included only those treated for 2 weeks (studies 1-4)
Beta coefficient indicates mean difference in parameter between groups
a. Variables adjusted for include the baseline value for creatinine peak or haemoglobin nadir; 5FC and sex for haemoglobin drop and nadir

Supplementary Table 3. Summary of toxicity management and reporting in published studies using AmBd in induction treatment of HIV-associated CM

Author, year	Country	n	*n* on AmBd	induction CM drug treatment	Nephrotoxicity prevention and management	Nephrotoxicity	Electrolyte loss	Anaemia	Early discontinuation AmBd
High income countries									
**de Lalla 1994**	Italy	31	31	AmBd 1mg/kg/d(±5FC) for 2 weeks	discontinue if creat>4mg/dl	23% grade III (1.9-3.4x ULN)	-	-	16%
**Sharkey 1996**	USA	55	17	ABLC v AmBd 0.7 mg/kg/d for 2 weeks, then 3x/week 1.2mg/kg for 4 weeks	Saline pre-loading and omit dose if creat >3, restart when <2.5mg/dl.	mean creat rise 0.7mg/dL at 2 weeks	24% had decrease in K and Mg (degree not specified)	mean drop in Hb 2.5g/dL at 6 weeks, 59% transfused	53%
**van der Horst 1997**	USA	381	381	AmBd 0.7mg/kg/d(±5FC) for 2 weeks	Saline pre-loading 'could be considered', discontinue if creat>3.5 ULN	1% Creat >3xULN	<1% hypokalaemia	1 case of haemolytic anaemia	3%
**Leenders 1997**	Holland	28	15	AmBd 0.7 v AmBisome 4mg/kg/d 3 weeks	Saline pre-loading. Miss 2 doses if creat >3x normal or 2x baseline	8% Creat >3xULN	31% K <3mmol/L(grade II)	46% Hb drop<2g/dL, mean 20% dec from baseline at 3 wks	13%
**Robinson 1999**	USA	236	236	AmBd 0.3-0.7mg/kg/d+5FC for 2 weeks	dose alt diem if creat >3mg/dl		-	-	-
**Hammill 2010**	USA	267	87	AmBd 0.7 v AmBisome 3 or 6mg/kg/d for 2 weeks	Saline pre-loading 'permitted'	33% creat >1.2mg/dL and 2x baseline value	30% K<3mmol/L	44% Hb<8g/dL	5%
Low and middle income countries									
**Joly 1996**	Burundi	90	44	AmB intralipid v AmBd 0.7mg/kg/d for 2 weeks, then 1mg/kg/d alt die 4 weeks	not described	creat >150mmol/L: 30% at 2 weeks, 50% at 6 wks	-	median Hb drop from 10.8 to 9.3g/dL at 6 weeks	5%
**Pittisuttithum 2001**	Thailand	106	106	AmBd 0.7mg/kg/d for 2 weeks	oral K supplements for hypokalaemia, dose reduction if creat rising	26% ' significant rise 'in creat	56% K<3.5mmol/L (grade I)	-	
**Tansuphaswadikul 2006**	Thailand	57	57	AmBd 0.7mg/kg/d for 1 or 2 weeks	not described	creat>2mg/dL 20% week 1, 14% week 2	K<3mmol/L 44% week 1, 31% week 2	median Hb drop 11.2 to 9g/dL in 1-week group and to 7.9g/dL in 2-week group	0

**Kambugu 2008**	Uganda	136	136	AmBd 0.7mg/kg/d for 2 weeks: historic cohorts 2001(n=92) and 2006(n=44)	Saline pre-loading in 2006. Dose alt die and give >2L saline if creat >3mg/dL	9% creat >3mg/dL at 2 weeks, median rise 0.6mg/dL	-	-	1%
**Pappas 2009**	Thailand/ USA	143	143	AmBd 0.7 (+Flu 400/800) 2 weeks	not described	not reported except no difference between arms	-	-	-
**Lightowler 2010**	SA	186	148	AmBd 0.7 or Flu 400 for 2 weeks	Saline pre-loading. If creat>220 µmol/l, early switch to fluconazole	11% creat>220umol/L	4% hypokalaemia (not defined)	-	11%
**Day 2013**	Vietnam	299	299	AmBd 1 mg/kg/d (±5FC/Flu 800): AmBd for 4 weeks, combination 2 weeks	Saline pre-loading	2% grade III/IV (all arms)	18% K<2.5mmol/L (grade III)	37% anaemia grade III/IV	8% stopped or dose modified
**Bahr 2014, Boulware 2014**	Uganda	142	142	AmBd 0.7-1mg/kg/d (+Flu 800) For 2 weeks	Saline pre-loading, daily supplementation oral K and Mg	10% grade III	Pre-emptive replacement decreased incidence of grade III hypokalaemia from 38% to 9%	50% grade III/IV	-
**This cohort**	Thailand/ Uganda/ Malawi/ South Africa	368	368	AmBd 0.7 or 1mg/kg/d ±5FC/vori/Flu/IFN for 5-14 days	Saline+20mmol KCl pre-loading, oral K and Mg in S Africa	9.5% creat >220umol/L(grade III), mean creat rise 49umol/L at 2 weeks	hypoK 4% grade III, 1% grade IV; hypoMg 25% grade III, 6% grade IV	16% grade IV, 4% tranfused, mean change -1.5g/dL at 7d and -2.3g/dL at 2 weeks	6%

Suppl Figure 1. Individual data points and fitted Loess curves for potassium values over the first 14 days of antifungal therapy

3a. All patients receiving 14 days' AmB-based induction therapy 3b. Plot by AmB duration short-course vs standard

Broken line indicates grade III DAIDS adverse event threshold of 2.5 mmol/L

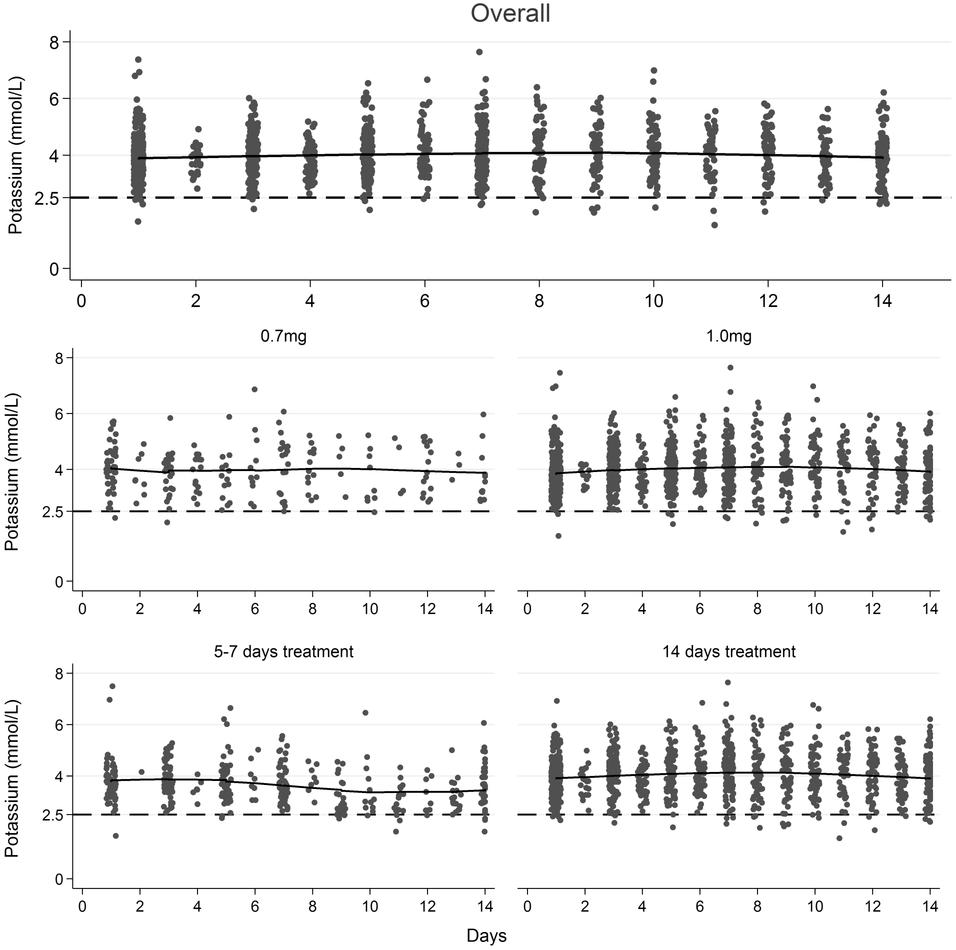

Supplement: Supplemental material [file AAC.01698-15_zac011154552so1.pdf]
